# Harnessing Antitumor CD4^+^ T Cells for Cancer Immunotherapy

**DOI:** 10.3390/cancers14010260

**Published:** 2022-01-05

**Authors:** Myriam Ben Khelil, Yann Godet, Syrine Abdeljaoued, Christophe Borg, Olivier Adotévi, Romain Loyon

**Affiliations:** 1 UMR1098, EFS BFC, INSERM, Interactions Hôte-Greffon-Tumeur/Ingénierie Cellulaire et Génique, University of Bourgogne Franche-Comté, F-25000 Besançon, France; Myriam.Benkhelil.ext@efs.sante.fr (M.B.K.); yann.godet@univ-fcomte.fr (Y.G.); Syrine.ABDELJAOUED@efs.sante.fr (S.A.); christophe.borg@efs.sante.fr (C.B.); olivier.adotevi@univ-fcomte.fr (O.A.); 2 Department of Medical Oncology, University Hospital of Besançon, F-25000 Besançon, France

**Keywords:** CD4^+^ T cells, cancer immunotherapy, immune checkpoint inhibitors, adoptive cell transfer, cancer vaccine

## Abstract

**Simple Summary:**

Diverse evidence revealed that CD4^+^ T cells play an important role in antitumor immunity by promoting or suppressing cytotoxic T cell responses. This review outlines the role of CD4^+^ T subsets within the tumor microenvironment and summarizes the latest progress regarding their potentials in cancer immunotherapy and methods for improving outcomes in cancer strategies by modulating CD4^+^ T responses.

**Abstract:**

Over the past decades, CD4^+^ T cells have been considered as a supporting actor in the fields of cancer immunotherapy. Until recently, accumulating evidence has demonstrated the critical role of CD4^+^ T cells during antitumor immunity. CD4^+^ T cells can either suppress or promote the antitumor cytotoxic CD8^+^ T cell responses, either in secondary lymphoid organs or in the tumor. In this review, we provide an overview of the multifaceted role of different CD4^+^ T cell subsets in cancer immune response and their contribution during cancer therapies. Specifically, we focus on the latest progress regarding the impact of CD4^+^ T cell modulation on immunotherapies and other cancer therapies and discuss the prospect for harnessing CD4^+^ T cells to control tumor progression and prevent recurrence in patients.

## 1. Introduction

Recent advances in cancer therapy have shown clear benefits targeting the immune system. Over the last decade, the discovery and understanding of regulatory molecules resulted in the development of Immune Checkpoint Inhibitors (ICI) therapy using antibodies blocking inhibitory receptors. Among the inhibitory checkpoints, cytotoxic T-lymphocyte-associated protein 4 (CTLA-4) and programmed cell death protein 1 (PD-1) expressed by T cells are the most studied and are promising targets for cancer immunotherapy. Following TCR engagement, the expression of CTLA-4 and PD-1 is induced on the surface of T cells resulting in a decrease in their activation, proliferation and antitumor cytokine production [1,2]. Consequently, inhibitory antibodies were developed to restore antitumor T cell functionality and rescue T cell exhaustion (discussed later). Furthermore, Adoptive Cell Transfer (ACT) using Tumor infiltrating lymphocytes (TILs) and engineered T cells expressing Chimeric Antigen Receptors (CAR) targeting tumor cells have emerged as revolutionary advances in cancer treatment [3,4]. Broadly, these therapeutic modalities consist in improving the quantity and quality of antitumor CD8^+^ cytotoxic T cell (CTL) responses, resulting in impressive gains in survival. Recently, the role of CD4^+^ T cells during antitumor response has become increasingly appreciated. According to the cytokine spectrum, T helper (Th) cells can be divided into four major lineages including Th1, Th2, Th17 and Tregs. These Th cells are plastic, and each lineage can be converted to another lineage [5]. As novel immunotherapies and immunotherapy combinations are developed, understanding the different roles of CD4^+^ T helper cells in antitumor response appears as a major challenge for the upcoming years. Thus, CD4^+^ T cell-based immunotherapy in combination with other therapies might represent an effective strategy to control tumor progression and recurrence. In this review, we summarize the role of different CD4^+^ T cell subsets and their contribution to antitumor immunity. We also highlight the critical role of CD4^+^ T cells during cancer therapies and discuss potential strategies harnessing CD4^+^ T cells to control tumor progression and improve outcomes in cancer immunotherapy.

## 2. Effector CD4^+^ T Cell Subsets in Cancers

It is clearly established that CTLs present ultimate effectors of tumor rejection. Indeed, tumor cells widely express Major Histocompatibility Complex (MHC)-I molecules necessary for CTLs to recognize specifically tumor antigens and exercise their cytotoxic activity. In contrast to the beneficial effects of CTLs in the vast majority of cancers, the effect of CD4^+^ T cell populations on clinical outcomes is not so clear. Moreover, with respect to MHC-I expression, tumor cells could also express MHC-II, which increases tumor recognition by the immune system and enhances CD4^+^ T cell immunity. It has been reported that the expression of HLA-DR molecules by tumor cells is associated with a good prognosis in cancer patients suggesting that direct recognition of tumor cells by CD4^+^ T cells could be beneficial [6]. More recently, in melanoma, MHC-II expression on tumor cells has been associated with improved progression-free survival (PFS) and overall survival (OS) after immunotherapy in melanoma (*n* = 23; PFS *p* = 0.02; OS *p* = 0.003) [7] (*n* = 166; PFS *p* = 0.0004; OS *p* = 0.0011) [8] (*n* = 181; OS *p* = 0.01) [9] and classic Hodgkin lymphoma patients (*n* = 50; *p* = 0.014) [10]. It was also associated with a higher number of both CD4^+^ and CD8^+^ TILs, an absence of lymphovascular invasion and increased formation of Tertiary Lymphoid Structures (TLS) with higher levels of IFN-γ, IL-2 and IL-12 mRNA (Th1 associated cytokines) in triple-negative breast cancer [11]. Interestingly, in addition to the indirect critical role of CD4^+^ T cells during antitumor response, effector CD4^+^ T cells are able to recognize and kill directly MHC-II^+^ tumor cells [12,13,14]. This CD4^+^ subset with cytotoxic activity has been recently further characterized in different human cancers by Jandus group [12].

**Figure 1:**
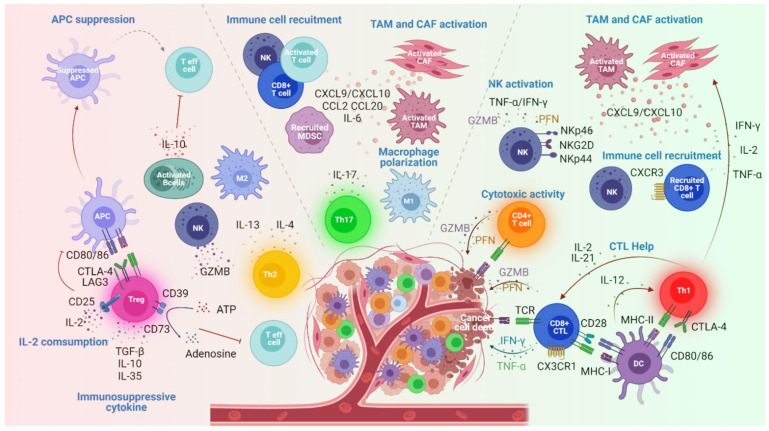
CD4^+^ T cell subsets in the tumor microenvironment (TME). Th1 cells (in red) exert a prominent antitumor activity. These cells produce various cytokines (IFN-γ, TNF-α, IL-2 and IL-21) to induce CTL help, CAF and TAM activation promoting immune cell recruitment such as CD8^+^ cytotoxic T lymphocytes (CTL) and Natural Killer (NK) cells that mediate tumor-killing activity. CD4^+^ T cells with cytotoxic activity (in orange) secrete GZMB and PFN and directly kill target cells. Th17 cells (in green) by producing IL-17 cytokine induce the polarization of M1 macrophage and the recruitment of antitumor immune cells (NK and CD8^+^ T cells) and Myeloid-derived suppressor cells (MDSC). Th2 cells (in yellow) present an ambivalent role in cancer. These cells contribute to antitumor responses by inducing NK cell activation (IL-4 production) and protumor responses by promoting M2 macrophage polarization (IL-4 production) and suppressive IL-10 producing-B cell activation (IL-13 production). Treg cell (in pink) presence within the tumor impedes antitumor responses by suppressing effector T cell activity through immunosuppressive cytokine production (TGFβ, IL-10 and IL-35), IL-2 consumption, antigen-presenting cell (APC) suppression and ATP- adenosine conversion. Tumor progression or regression depends on the overall effect of the complex cellular network within TME. DC, dendritic cell; CAF, cancer-associated fibroblasts; TAM, tumor-associated macrophage; GZM, granzyme; PFN, perforin; TNF-α, tumor necrosis factor-α.

CD4^+^ T cell subsets have varied impacts on tumor growth while they play a pivotal helper role in orchestrating cancer immunity (Figure 1, Table 1). All various subsets of effector T cells may be found in the tumor microenvironment (TME) including Th1, Th2, Th9, Th17, Tregs, CD4^+^ CTL and T follicular helper cells (Tfh) and can be located in the core of the tumor and the invasive margin or the adjacent TLS forming the tumor immune contexture. These CD4^+^ T cell subsets, considered as good or bad immune cells, present diverse functions, sometimes opposite, in antitumor immunity [15].

### 2.1. Th1 Cell Subsets

Th1 cells are mainly characterized by the expression of the transcription factor T-bet, the chemokine receptors CXCR3 and the production of IFN-γ, TNF-α and IL-2. Early reports have described the critical role of Th1 subset tumor infiltration and how Th1 gene signatures were associated with good clinical outcomes in patients with melanoma [51], breast [52,53], ovarian [54] and colorectal cancers [55,56,57,58,59]. More recently, we have reported that in NSCLC patients, higher levels of peripheral blood circulating telomerase (hTERT)-specific Th1 cells are associated with better prognosis [60]. Indeed, tumor-antigen specific CD4^+^ T cells with Th1 polarization can indirectly promote tumor rejection by cytokine production to support survival and stimulate CTL and Natural Killer (NK) cell functions into the tumor [16,17,18]. Moreover, the production of IFN-γ by Th1 cells is associated with a better PFS [59,61,62,63] playing a key role in enhancing MHC-I and II expression by tumor cells and upregulating tumor-derived antigen presentation pathways [19,20]. Additionally, IFN-γ production by Th1 cells induces secretion of CXCL9 and CXCL10 by tumor-associated macrophages (TAM) and cancer-associated fibroblasts (CAF) as well as tumor cells present in the TME that promote tumor-effector T cell infiltration in melanoma patients [21,22]. Thus, Th1 cells indirectly shape an optimized immune microenvironment in the tumor [16,64]. Furthermore, in a recent study, Zander et al. highlighted the critical role of IL-21 produced by CD4^+^ T cells in the generation of a distinct subset of CX3CR1 cytotoxic CD8^+^ T cells with a potent cytolytic function in a melanoma mouse model. Hence, CD4-IL-21/CD8-CX3CR1 pathway is operational during tumorigenesis and could be used therapeutically to enhance CD8^+^ T cells killer functions over tumor progression [65].

### 2.2. Th2 Cell Subsets

Th2 cells are characterized by the expression of the master transcription factor GATA-3, the chemokine receptors CCR3 and CCR4 and the production of cytokines such as IL-4, IL-5 and IL-13. Ambivalent roles have been ascribed to Th2 cells in the context of cancer as well. Indeed, their presence in TME can be either beneficial or detrimental for patient survival [61]. Th2 cell infiltration has been associated with poor outcomes in ovarian and pancreatic cancer [24], while Th2 cells have been associated with favorable outcomes in Hodgkin’s lymphoma and breast cancer [66,67] and OVA-specific Th2 cells elicited a long-lasting antitumor response in transgenic mice [25]. Moreover, in B-cell lymphoma model, the induction of tumor-specific Th2 inflammatory immune response induces M2-type macrophages infiltration and cancer eradication [23]. The detrimental role of Th2 cells infiltration in cancer could be associated with the activation of B cells to produce IL-10 resulting in suppressive effects, whereas IL-4 production by Th2 memory cells [27,28] could stimulate NK cells promoting their antitumor cytotoxic activity through perforin and granzyme B production [24,25,26].

### 2.3. Th9 Cell Subsets

Th9 cells are the most recently identified subset among effector CD4^+^ T cell subsets [68]. Indeed, prior to their recognition as an individual subset, Th9 cells were once believed to be included within the Th2 cell subsets. Naïve T cells are differentiated into Th9 cells in the presence of TGFβ and IL-4, mostly secreted by Th2 cells. Th9 cells are characterized by the expression of transcription factors IRF4 and PU.1 and the production of high level of IL-9 [69]. The direct contribution of Th9 cells in cancer remains debated. Indeed, it has been shown that IL-9 production promotes uptake and antigen presentation by DC to CD8^+^ T cell activation [29]. Further studies on melanoma mice models have demonstrated that Th9 cell subsets promote antitumor activity [70,71] and were found to be less exhausted and fully cytolytic in TME [72]. More recently, Sek et al. have shown in a mouse model that adoptive transfer of tumor-specific Th9 cells can eradicate tumor cells [30]. In contrast, a high level of IL-9 was detected in the sera of 18 out of 44 patients with Hodgkin’s lymphoma and has been associated with poor prognosis [73]. In lung cancer mouse model, Salazar et al. have reported that Th9 cells promoted tumor progression and metastasis and that enhanced numbers of Th9 and Th17 cells in patient lung cancer tissue correlated with poor survival [31]. Thus, much more effort will be required for a better understanding of Th9 cell development and function in the tumor context.

### 2.4. Th17 Cell Subsets

The differentiation to Th17 subsets is governed by transcription factor RORγt and is characterized mainly by the production of IL-17 and IL-22 cytokines and the expression of CCR6. Unlike Th1 cells, Th17 cells were associated either with poor or with good prognosis. Indeed, various reports have associated the infiltration of Th17 cells to poor prognosis in colorectal [38], lung [39], pancreatic cancers [74] and hepatocellular carcinoma [36,75]. However, Th17 cells were associated with a better clinical outcome in some esophageal [76,77], prostate [78], gastric [74,79] and uterine cervical cancers [80]. This controversial role of Th17 subsets may be associated with the secretion of IL-17, which plays dichotomous roles in both cancer growth and tumor elimination. Indeed, studies have shown that IL-17 stimulates tumor cell proliferation in breast and ovarian cancers [81,82] and promotes prostate cancer invasion and hepatocellular carcinomas metastasis [36,83]. In addition, IL17 production promotes angiogenesis in colorectal and lung cancers via stimulating vascular endothelial growth factor (VEGF) produced by cancer cells [38,39].

Indirectly, IL-17 contributes to myeloid-derived suppressor cells (MDSCs) recruitment in lymphoma, prostate and melanoma models [37]. In contrast, IL-17 appears tumor protective and contributes directly and indirectly to antitumor function by increasing antitumor responses, thus resulting in tumor regression [40]. It has been reported that IL-17 receptor ligation signals apoptosis in breast cancer cells [84]. Moreover, IL-17 production enhances antitumoral macrophage polarization [32] and CD107a, TNF-α, IFN-γ, perforin, NKp46, NKG2D and NKp44 expression in NK cells [33]. Similarly, IL-17 suppresses cancer progression via increasing IFN-γ^+^ T cell activity in colon cancer models [34]. Ultimately, IL-17 instructs indirectly innate and adaptive antitumor immune systems to become cytotoxic by stimulating IL-6, CCL2, CCL20, CXCL9 and CXCL10 production that recruits and activates T cells, dendritic cells (DCs) and NK cells [32,85].

### 2.5. Treg Cell Subsets

Immunosuppressive natural Treg subpopulation originated from the thymus (tTreg). However, conventional naïve CD4^+^ T cells can be converted into Treg cells (pTreg) in an IL-2 and TGFβ-rich environment.

Tregs have been detected in circulation and TME and can be potently suppressive in breast cancer patients [35,86] by inhibiting antitumor immunity [87,88,89], resulting in a poor prognosis [90]. Their suppressive activity is maintained through the TCR stimulation by MHC-II molecules [91] and can be assured by the production of immunosuppressive cytokines such as IL-10, TGFβ and IL-35 in the TME. Immunosuppressive receptors expression on Treg cells such as LAG3, TIGIT and/or CTLA-4 also inhibit antitumoral effector T cells [41,92]. In addition, the expression of high-affinity IL-2 receptor on Tregs assures another immune-suppressive mechanism with the consumption of IL-2 present in the TME resulting in its low availability [42]. Other immune-suppressive mechanisms for Tregs have been described and reviewed [93]. Interestingly, tumor-specific Tregs have not only been identified in TILs but also in peripheral blood [94,95,96,97], and their TCR specificity may have potential implications for cancer immunotherapy, as has been highlighted by Rosenberg’s group [98]. A poor outcome has been reported in 188 patients with liver metastasis colorectal cancer presenting a high number of Tregs in TILs [99]. Recently, Osman and colleagues have reported in mice with polyposis that TCF-1-deficient Treg cells strongly suppressed T cell proliferation and cytotoxicity and promoted tumor growth [100]. Moreover, in the same study, it has been shown that tumor-infiltrating Treg cells have significantly lower expression of TCF7 compared to adjacent normal tissue and PBMCs and that Treg cell-specific TCF-1 expression differentially regulates TH17-mediated inflammation and T cell cytotoxicity, which can determine colorectal cancer outcome in patients.

However, the prognostic impact of Tregs in cancer patients is still controversial. Indeed, studies in colon and ovarian cancers have associated Tregs infiltration with a better prognosis [101,102,103,104,105,106]. Saito et al. suggested that this dichotomous role of Tregs could be explained by the presence of different subsets of tumor-infiltrating FoxP3+ Tregs including FoxP3^high^ and FoxP3^low^ Tregs. In fact, FoxP3^high^ Tregs have been identified as suppression-competent, while FoxP3^low^ T cells secreting inflammatory cytokines have been identified as non-suppressive Tregs in colorectal patients [107]. Furthermore, in a meta-analysis including 17 published studies with 3811 CRC patients, authors have shown that high density FOXP3+ Tregs within the tumor, especially at the stromal compartment, results in a favorable outcome, highlighting the importance of Treg density as well as localization inside the TME [106].

Of note, these contradictory observations connecting Tregs and clinical outcomes can be also partially explained by the markers used to detect Tregs such as CD25 and FOXP3 [107], which are not fully restricted to Tregs in humans. Altogether, these observations suggest that a better classification and identification of Treg cells are required to associate Tregs and patient outcomes.

### 2.6. CD4^+^ CTL

CD4^+^ CTLs were first identified in alloreactive responses against gene products coded by MHC of mice-H2 complex [108]. As every CD4^+^ T cell subsets, CD4^+^ CTLs recognize antigen peptide in the context of MHC-II [109]. This population was also described under chronic viral infections in mice such as gamma-herpes virus [110] and also in human such as human cytomegalovirus [111], human immunodeficiency virus 1 [112] and hepatitis virus [113]. Several reports have described CD4^+^ CTL generation in vitro. These cells could be derived from Th0 [114], Th1 [111] and Th2 [115], Th17 [116] and Treg [117] effector subsets in a viral infection context. Available evidence suggests that CD4^+^ CTL express RUNX3, BLIMP-1 and EOMES transcription factors in TME while T bet expression remains not clear depending on specific conditions and disease [118]. The earliest reports that CD4+ T cells could be directly cytotoxic to tumor cells have been shown in the context of chemotherapy-induced lymphopenia [119]. Indeed, the engagement of OX40, a costimulatory molecule expressed primarily on activated CD4+ T cells, induces tumor-specific CD4+ T cell population able to eradicate advanced melanomas in mice and also human melanoma cells in an in vitro model. More recently, studies have shown the contribution of CD4^+^ CTL in cancer cell eradication in human using RNAseq in multiple solid cancers (non-small-cell lung cancer [120], colorectal cancer [43], hepatocellular carcinoma [44], bladder cancer [45], osteosarcoma, breast cancer [46,47], head and neck cancer [48] and melanoma [12]). Indeed, it has been documented that CD4^+^ CTL express cytolytic effector molecules such as granzymes, perforin and other granule-associated proteins such as NKG7 and granulysin, both in the tumor and in the circulation of patients.

### 2.7. Tfh Cell Subsets

A smaller population of CD4^+^ T cells corresponds to Tfh cells. These cells express transcription factor BCL6 and surface markers CXCR5, ICOS and PD-1 and produce IL-21 and IL4 [49]. In non-cancer context, these cells play an important role during germinal center (GC) formation and present a crucial source of cytokines essential for GC expansion and B cell isotype class switching. In human cancers, the presence of TLS and the enrichment of Tfh and B cells correlate with prolonged survival and favorable therapeutic response in melanoma (*n* = 39; PFS *p* = 0.003) [121], breast (*n* = 794; PFS *p* = 0.0036) [122] and lung cancers (*n* = 478; OS *p* = 0.0013) [123]. Interestingly, a recent study in a mouse model has shown that tumor-specific Tfh-B cells interactions as well as IL-21 production result in antitumor immunity by enhancing granzyme B expression by tumor-infiltrating CD8^+^ T cells [123].

Altogether, CD4^+^ T cell subsets appear as critical players in TME remolding. Furthermore, based on immunology and clinical knowledge accumulated over the last decade, the interest in immunotherapy to restore a Th1 microenvironment may produce tangible benefits for cancer eradication.

## 3. CD4^+^ T Cell Help for Cancer Immunotherapies

### 3.1. Cancer Vaccines

**Figure 2:**
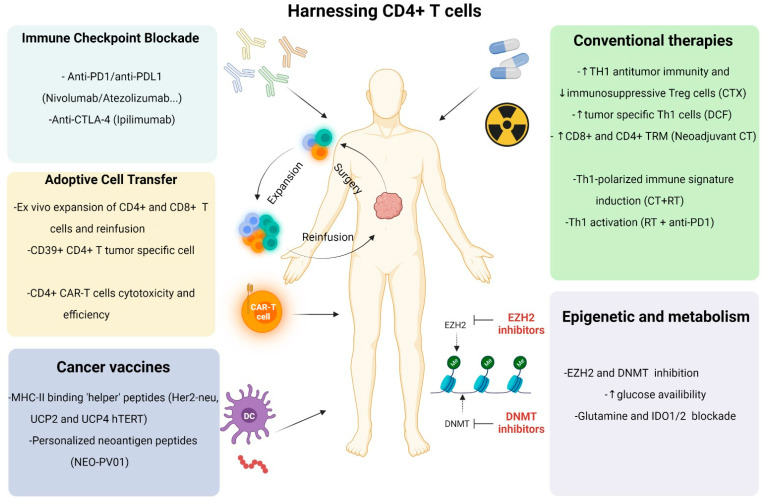
Harnessing CD4^+^ T cells to improve cancer therapy. While CD4^+^ T cell subsets function during antitumor response has become more appreciated, cancer therapy strategies might benefit from including approaches to modulate CD4^+^ T cell subset. Conventional therapies, chemotherapy (CT) and radiotherapy (RT) have an impact on CD4^+^ T cell response modulation. Cyclophosphamide (CTX) results in the development of robust Th1 antitumor immunity and immunosuppressive Treg cell depletion. Tumor-specific Th1 subset increases after DCF (Docetaxel, Cisplatin and 5-fluorouracil) regimen. Neoadjuvant CT promotes antitumor CD8^+^ and CD4^+^ T Resident Memory (TRM) cells. The combination of CT and RT induces Th1 polarized immune signature while RT and anti-PD1 immunotherapies result in Th1 cell activation. Immune checkpoint blockade (ICB) relies on blocking antibodies able to inhibit immune checkpoint receptors axis such as PD-1/PD-L1 ((Nivolumab/Atezolizumab) and/or CTLA-4 (Ipilimumab), which provide effector CD4^+^ T cell expansion resulting in efficacious CD8^+^ T cell response. Adoptive cell transfer of T cells includes tumor-infiltrating lymphocytes (TILs) transfer and genetically engineered T cells transfer. Both strategies should include CD4^+^ T cells to provide help at the tumor site. Thus, transferred ex vivo expanded TILs (CD4^+^ and CD8^+^ T cells) or Chimeric Antigen Receptor (CAR) T cells enhance antitumor responses. Peptides used in therapeutic cancer vaccines including MHC-II epitopes (Her2-neu, UCP2 and UCP4 hTERT, NEO-PV01) are able to activate CD4^+^ T cells and rely on help signals to CD8^+^ T cells. Targeting epigenetics regulators such as EZH2 and DNMT markers could alter Treg functionality and induce antitumor Th1 responses. Otherwise, targeting metabolism programming (glucose, glutamine, Indoleamine 2,3-dioxygenase IDO…) could boost effector T cell response and inhibit Treg polarization. EZH2, enhancer of zeste homolog 2; DNMT, DNA methyltransferase 1.

To take advantage of the specificity and cytotoxic capacity of CD8^+^ T cells, many cancer immunotherapies including cancer vaccines were designed [50] (Figure 2). This therapeutic strategy aims to stimulate tumor-specific T cells to regain control over tumor growth and induce its regression. Likely barriers to efficacy involve peripheral tolerance and immunosuppressive TME limiting the generation of effective vaccine-specific CTL. Hence, studies have reported disappointing outcomes when targeting CD8^+^ T subsets in cancer vaccine strategy [124]. As mentioned above, CD4^+^ T cells play a crucial role in supporting effective antitumor CTL response. For this reason, therapeutic vaccines include MHC-II binding “helper” peptides [125]. Furthermore, Ahrends et al. have specified the impact of the CD4^+^ T cell’s help signal on CTL at the molecular level in the antitumor vaccine mouse model [126]. Thus, CD4^+^ T cells help initiate a gene expression program in CD8^+^ T cells that enhances CTL function by improving their migratory potential and downregulating the expression of their co-inhibitory receptors resulting in increased antitumor efficacy of cancer therapeutic vaccination. Moreover, this help generates optimized CTL memory with easier recall capacities [127]. Of note, results are divided regarding the tumor-specificity of CD4 help. Indeed, Bos and Sherman have demonstrated that specific CD4^+^ T cells promote tumor eradication compared to non-tumor-specific CD4^+^ help during CD8^+^ priming [16]. In contrast, Ahrends et al. have reported in a mouse model that vaccination including tumor-unrelated helper epitopes increased CTL priming, effector and memory T-cell programming [126,128]. In this context, several cancer-vaccine approaches targeting CD4^+^ T cell response have offered promising results in clinical trials [129]. In breast cancer patients, Her2/neu helper peptides administered as peptide-pulsed type 1 DC vaccine induced durable specific CD4^+^ T cell responses [130] and complete regressions [131,132]. In melanoma, patients receiving pulsed DC vaccine including CD8 epitopes and helper peptides develop CTL and helper T cell responses with better clinical outcomes compared to patients vaccinated only with CD8 epitopes. Similarly, helper epitopes have demonstrated efficacy in the treatment of HPV-induced pre-malignant lesions [133,134].

hTERT overexpression has been reported in various cancers, and several hTERT helper peptide vaccines have been reported [129,135,136,137,138]. Moreover, we described highly promiscuous HLA-II binding hTERT derived peptides called universal cancer peptides (UCP). These peptides were able to stimulate strong Th1 immunity, which help the generation of potent memory antitumor CD8^+^ T cells in preclinical studies [139,140,141]. This UCP-based therapeutic anticancer vaccine is clinically evaluated as monotherapy in metastatic NSCLC patients (NCT02818426) and glioblastoma (NCT04280848).

Notwithstanding this great progress in cancer vaccine development, the Food and Drug Administration (FDA) approved the only DC-focused cell-based vaccine, sipuleucel-T, for prostate cancer patients more than 10 years ago [142]. Interestingly, the implementation of other immunotherapies such as Immune Checkpoint Inhibitors (ICI) has revolutionized cancer therapy by improving survival rates and observing potential cures (discussed later). Consequently, therapeutic cancer vaccine approaches were re-emerging to increase response rates in combination with ICI. Thus, we have initiated UCP cancer vaccine combined with ICI approaches for clinical trials in Human Papillomavirus (HPV) positive cancers (NCT03946358) and NSCLC (NCT04263051) [143]. In a larger recent study (NCT02897765) in patients with advanced melanoma (*n* = 34), non-small cell lung cancer (*n* = 27) or bladder cancer (*n* = 21), a personalized neoantigen-based vaccine, NEO-PV-01, in combination with PD-1 blockade induced the development of neoantigen-specific CD4^+^ and CD8^+^ T cell responses post-vaccination in all patients [144]. Moreover, in recent melanoma clinical trials, neoantigen vaccination induces CD4^+^ T cell responses and improves patient survival (NCT02035956, *n* = 15) [145], (NCT02287428, *n* = 56) [146,147].

Overall, therapeutic vaccination incorporating CD4^+^ T cell target could be an efficient approach in various cancers, especially when combined with ICI therapy. Nevertheless, one major limitation of cancer vaccine design based on MHC-II epitopes is the identification of the ideal epitope to target. Indeed, current tools used for MHC-II peptide binding display low accuracy because it is difficult to determine the core binding region of MHC-II-ligands when MHC-II are highly polymorphic and the size of the peptides presented varies [148]. Further studies are needed to improve epitope predictions and explore additional MHC-II restricted epitopes.

### 3.2. Immune Checkpoint Inhibitors

ICI have revolutionized the treatment paradigm of several cancer types and is currently the most successful and widely used form of cancer immunotherapeutic strategy. It consists of the administration of blocking antibodies to inhibit immune checkpoint receptors axis such as PD-1/PD-L1 and/or CTLA-4. The first ICI receiving FDA approval in 2011 was Ipilimumab, an anti-CTLA-4 for patients with advanced melanoma. Then, the FDA approved the first anti-PD-1 in 2014, pembrolizumab, as treatment of metastatic melanoma, resulting in remarkable progress in cancer treatment. Since then, nivolumab as PD-1 inhibitors and atezolizumab, durvalumab and avelumab as PD-L1 inhibitors have been approved for the treatment of patients with several cancers [2,149,150].

Initial studies reported that tumors sensitive to anti-CTLA-4 treatment have shown a dependence on CD4^+^ T cell population [151,152,153]. As CTLA-4 molecules are expressed by both CD4^+^ effector T cells and Tregs, this dependence to the CD4^+^ T subset could be explained by the role of help assured by CD4^+^ T cells or the effect of the depletion of the Treg population [154,155]. Nevertheless, CD4^+^ T cell activation after CTLA-4 blockade in tumors has been recently reported [156]. It has also been shown that anti-CTLA-4 and anti-PD-1 combination therapy resulting in increasing Th1-like CD4^+^ effector T cells frequency [157]. These various pieces of evidence have challenged the conventional view of ICI reinvigorating CD8^+^ T cells supporting the important function of CD4^+^ T cells in antitumor response. Moreover, Jiao et al. observed an enhanced Th1 subset signature in paired pre- and post-treatment primary soft-tissue prostate tumors in patients treated with ipilimumab [158]. Nevertheless, patients with prostate cancer bone metastasis ipilimumab-treated presented an increase in Th17 cells but not Th1 cells comparing pre-treatment and post-treatment. These data suggest that the distinct TME with a higher TGFβ in the osseous environment than the non-osseous one can restrain Th1 polarization and expand CD4^+^ T cells toward a Th17 lineage. Consistent with this hypothesis, the combination of anti-TGFβ and anti-CTLA-4 significantly increased the frequency of Th1 cells and decreased the frequency of Treg, underlying the role of the TME and CD4^+^ T cells in ICI treatment antitumor efficacy [158].

Thus, CD4^+^ T cell responses might be required systemically to achieve efficacious CD8^+^ T cell responses under ICI therapy. In the last years, studies have investigated deeper the role of systemic CD4^+^ T cells immunity in therapeutic efficacy. Various studies have reported that peripheral blood CD8^+^ T cells proliferate after ICI treatment [159,160,161]. Similarly, preclinical studies have demonstrated the importance of systemic CD4 immunity in immunotherapy efficacy and that CD4^+^ T cell proliferation is correlated with tumor rejection in murine models [159,162,163]. In melanoma, CD4^+^ PD-1- CD127^low^ T cells subset, suggesting unexhausted cells, increased in responders treated with ICI compared to non-responders [162]. In addition, recent reports have shown that systemic CD4^+^ T cells influence positively ICI outcomes [160] and that a repertoire of pre-existing blood CD4^+^ T cells predicts better clinical outcomes in melanoma patients treated with anti-CTLA-4 [161]. More recently, Kagamu et al. demonstrated that a high level of functional systemic CD4^+^ T cells before anti-PD-1 therapy correlates with PD1^+^ CD8^+^ T cells in patients with NSCLC [164].

Interestingly, extensive phenotypic characterization of CD4^+^ T cell subset has identified several states of cytotoxic CD4^+^ T cells expressing cytolytic effector proteins. These CD4^+^ T cells with cytotoxic functions have been found in certain viral infections [165,166], autoimmune disorders [167] and cancer. In transitional cell carcinoma, a specific type of bladder cancer, Oh et al. identified multiple states of cytotoxic CD4^+^ TILs able to kill tumor cells in an MHC-II dependent fashion. Moreover, the predictive value of cytotoxic CD4^+^ T cell specific signature has been reported in a large cohort of metastatic melanoma patients treated with anti-PD-1 [45]. A deeper characterization of these population in various human cancers have shown the involvement of SLAM family member 7 (SLAMF7) gene expression as a direct regulator of tumor-specific CD4^+^ T cell cytotoxicity [12]. In addition, Nagasaki’s group has recently highlighted, in classic Hodgkin lymphoma, the critical role of cytotoxic CD4^+^ T cells in both spontaneous and PD-1 blockade-mediated antitumor immunity in MHC-II-expressing tumors, even without MHC-I expression [168]. Interestingly, the combination of LAG-3 blockade with PD-1 blockade exhibited a stronger antitumor efficacy than either treatment alone, suggesting that LAG-3 could be a potential therapeutic target for combination therapies with PD-1 blockade in MHC-II expressing cancers [168]. Thereby, it is important to develop strategies targeting new immune checkpoints expressed by CD4^+^ T cells that can improve antitumor immunity as either monotherapies or combination therapies.

### 3.3. Adoptive Cell Transfer

The most successful cancer therapeutic strategy after ICI is the ACT of T cells (Figure 2). It includes TILs transfer and genetically engineered T cells transfer. Several studies have shown the association between ACT and high rates of durable responses and tumor regression in patients with melanoma [169,170], lymphoma [171,172] and epithelial cancers [173]. The majority of studies concerning ACT focus on the antitumor activity of CD8^+^ T cells. Nevertheless, due to accumulating recent evidence on the critical role of CD4^+^ T cells during antitumor responses, their implication in the ACT is being further explored [174].

#### 3.3.1. TIL Infusions

ACT using antigen-specific TILs is a personalized cancer treatment based on isolation of autologous CD4^+^ and CD8^+^ T cells from the tumor, ex vivo expansion and selection before reinfusion back to the patients to eliminate cancer cells. This concept has been initiated in 1980 by Rosenberg’s group and has shown sustained clinical efficacy. Indeed, large doses of IL-2 accompanied by autologous lymphokine-activated killer cells were effective when administrated to patients with metastatic melanoma [175]. Thereafter, the improvement of this approach with IL-2 expanded TILs showed more therapeutic potency [176,177]. Hence, the rationale to use TILs as a therapeutic option for advanced cancer patients has been provided.

Animal studies have shown that the combination of tumor-specific CD8^+^ and CD4^+^ T cells enhances significantly antitumor response [178,179]. While CD8^+^ T cells are viewed as the main effector cells in therapeutic approaches, a newly emerged concept of CD4^+^ ACT has shown promising results [23,180,181,182]. Indeed, complete melanoma regressions have been observed following infusion of antigen-specific CD4^+^ T cells [180,182]. Tumor-specific Th2 ACT efficiently eradicates cancer cells and confers long-lasting immunity in subcutaneous melanoma and B-cell lymphoma models [23]. In addition, tumor regression has been observed after ACT of TILs enriched with neoantigen-specific CD4^+^ T cells in cholangiocarcinoma patients [173]. Interestingly, the phenotypic characterization of these reactive T cells has revealed that these cells were predominantly effector memory CD4+ T cells with cytolytic potential; nonetheless, CD4 T cell helping CD8 or innate cells could not be excluded. More recently, CD39 has been identified as a marker for tumor-specific CD4^+^ T cell population in human TILs suggesting that CD39 could be used to identify, isolate and expand tumor-reactive T cell population before ACT immunotherapy [183]. Since another team has demonstrated that this tumor-specific CD39^+^ CD4^+^ TILs population expresses CXCL13, PD-1 and TOX exhaustion makers and this population could contribute to tumor-specific CD8^+^ T cell proliferation and DC maturation through in situ reactivation with PD-1 blockade [184]. In addition to clinical and therapeutic success of TILs, several cytokines were used to enhance ACT efficacy and overcome immunosuppressive TME. IL-2 has been used for more than 20 years to promote the growth of tumor-reactive T cells and enhance proliferation and survival of transferred TILs. However, it has been shown that this cytokine can also promote tumor progression [185] and Tregs expansion [186] and cause severe toxicity after infusion [187]. In contrast, it has been reported that IL-7 was greater in enhancing antitumor responses [188] and that IL-7 monotherapy resulted in expansion of CD8^+^ T cells and CD4^+^ Th cells but not Tregs [189]. Moreover, co-administration of IL-7 with tumor-reactive CD4^+^ T cells in a mouse model promotes their expansion, persistence and antitumor activity, corroborating the potential use of IL-7 as an adjuvant for CD4^+^ T cell-based ACT [190].

Despite many promising beneficial effects, TIL therapy has its limitations. Actually, TIL infusion therapy is the ultimate personalized immunotherapy since each patient needs its specific infusion product, which may be too long for some patients with rapidly progressive disease. Overall, TIL therapy presents a great anticancer therapy especially in melanoma and, in the future, possibly in other solid tumors.

#### 3.3.2. CAR-T Cell Therapy

Hereafter, the adoptive transfer of highly selected tumor-reactive T cells directed against overexpressed self-derived differentiation antigens in metastatic melanoma patients resulted in tumor regression due to the persistent clonal population of T cells [191]. Thus, using genetically manipulated T cells to target specific antigens could be a potential option in ACT therapy. Currently, there are two types of genetically modified T cells approaches: chimeric antigen receptor (CAR)-T cells and T-cell receptor (TCR)-engineered T cells.

Several clinical trials have shown the effectiveness of CAR-T cell therapy with controllable and tolerable toxicity and promising patient outcomes in some hematological malignancies [171,172,192,193,194]. Among CAR-T cell therapy for hematological cancers, CD19 CAR-T cells are the most prevalent and effective against ALL and B cell lymphoma CLL [195]. Recent studies have documented the influence of the CD4^+^/CD8^+^ ratio that might affect the antitumor capability of CAR-T therapy. Indeed, the balance between CD4^+^ and CD8^+^ CAR-T populations can positively influence antitumor reactivity, and B-ALL patients could achieve high remission rates [196,197,198]. Therefore, CAR-T cell therapy efficacy depends not only on generating a sufficient number of modified T cells but also on producing a defined phenotype and a specified CD4^+^/CD8^+^ ratio within the modified T cells [199]. Furthermore, the cytotoxic activity of CD8^+^ and CD4^+^ CAR-T cells and their capacity to eradicate tumor cells have been evaluated [200]. Indeed, Yinmeng et al. have demonstrated the loss of CD8^+^ CAR-T efficacy in mice due to T cell exhaustion. In contrast, CD4^+^ CAR-T presented equivalent cytotoxicity compared to CD8^+^ CAR-T associated with retention of efficacy in leukemic models [200]. Recently, in an interesting in vivo generation of CD19-CAR-T cell therapy approach, Agawarl et al. demonstrated that CD4-targeted lentiviral vector (CD4-LV) exhibited faster and superior tumor cell killing compared to CD8-LV alone or as a mixture with CD4-LV, suggesting higher CD4^+^ CAR-T cell efficacy mainly because CD8^+^ T cells are more prone to exhaustion [201]. In addition, they demonstrated that CD19^+^ B cell elimination was accompanied by CAR-T cells displaying a Th1/Th2 phenotype. Moreover, in solid tumors, Wang et al. demonstrated that CD4^+^ CAR-T cells mediated CD8-independent glioblastoma eradication and maintained long-term efficacy both in vitro and in vivo [202]. Similarly to Agawarl’s study, T cell exhaustion and subsequent effector dysfunction occurred more rapidly in the CD8^+^ T cells. Indeed, the longer effector potency of CD4+ T cells could be explained by the lower susceptibility of these cells to exhaustion in addition to their extensive capacity to produce more IFN-γ and TNF and to proliferate upon cancer cells challenge in a mouse model [196]. Likewise, a recent study from Rath et al. investigated TCR-engineered T cells in vitro and in vivo strategies to redirect CD4^+^ T cells against class-I tumor-associated antigens (TAA) epitopes and enhance transgenic CD8^+^ T cell function [203]. Thus, transgenic CD4^+^ T cells co-expressing class I–restricted tumor antigen–specific TCR and CD8αβ have shown comparable cytotoxic properties to engineered CD8^+^ T cells and were most potent by activating multiple transcriptional programs associated with enhanced antitumor function.

Otherwise, CD26 has been described as another interesting marker on human CD4^+^ T cells identifying a critical antitumor population [204]. Based on this, authors recently demonstrated in NSG mice that CD4^+^ CD26^hi^ CAR-T cells mediate durable and relapse-free immunity while bulk CD4^+^, Th1, Th2 or Th17 cell therapies elicited only transient delays in human tumor growth. In addition, they demonstrated that the co-transfer of CD8^+^ CAR-T cell is dispensable for the curative response [205]. In addition to their direct cytotoxic activity, CAR-T cells exercise additional mechanisms in the TME for optimal activity. More recently, Boulch et al. revealed that IFN-γ produced by CD4^+^ CAR-T cells results in IL-12 production that supports host immune and CAR-T cell responses more efficiently compared to CD8^+^ CAR-T cells [199]. Moreover, it has been reported that overexpression of T-bet transcription factor could enhance antitumor activity of CD4^+^ CAR-T cells [206].

Unlike effective CAR-T therapy in hematological malignancies, limited antitumor activity has been observed in solid tumors due to the lack of tumor-specific antigens expression, short-term persistence or insufficient CAR-T cells tumor infiltration. Indeed, to efficiently infiltrate tumor lesions, CAR-T cells must overcome immunosuppressive elements to elicit an efficient specific cytotoxic antitumor response. Thus, increasing the likelihood of successful delivery of help signals by modulating the tumor microenvironment was investigated. CAR-T cells engineered to release IL-12 were successfully used in preclinical models [207]. The therapeutic effect of IL-12 is to orchestrate Th1 antitumor immune response by increasing IFN-γ production by T and NK cells. Thus, CAR-T cells engineered to release IL-12 are more proliferative and cytotoxic and less apoptotic when applied to immunosuppressive ascites tumors [208]. Increased survival rates, a longer CAR-T cell persistence and higher systemic IFN-γ serum levels were reported in in vivo models with ovarian cancer [207].

Moreover, inducible IL-12 expression induces T cell infiltration and persistence within tumors and boosts the function of CAR-T cells targeting glypican-3 expressed by hepatocellular carcinoma by increasing IFN-γ production [208]. Thus, CD4^+^ T cell “help mimicry” in CAR-T cells and could result in increases in efficacy of this immunotherapeutic approach. T cells engineered with other cytokines are currently under investigation as well. Of note, studies have recently reported that CAR-T cells releasing IL-18 tip the balance within the tumor microenvironment toward Th1 acute phase response, thereby reducing the number of immunosuppressive Tregs and enhancing antitumor immunity [209,210]. It is also expected that the combination of CAR-T therapy with anti-PD-1 therapy will increase the efficacy of cancer immunotherapy in hematological malignancies [211] and solid tumors [212].

Overall, it appears that the CAR-T cell approach is less suitable for solid tumors and still needs optimization. Indeed, using mesothelin-redirected CAR-T cells in pancreatic cancer and a continuous antigen exposure model, Carl June’s team has recapitulated hallmark features of T cell exhaustion and described a T-to-NK-like T cell transition for both CD8 and CD4 CAR-T cells resulting in a dysfunctional phenotype [213]. Otherwise, it has been reported that CAR-T cell therapy induces the release of excessive amounts of cytokines such as IFNγ and TNF following T cell activation resulting in cytokine release syndrome thus multi-organ dysfunction [214]. Consequently, approaches of “safety switches” based on caspase 9 activation for rapid cell death induction have been used [215]. Another limitation of CAR-T cells is their recognition of only cell surface proteins. Indeed, CAR-T cells almost target tumor-associated antigens since surface tumor-specific antigens are rarely expressed, which represents a risk of cross-reactivity with normal tissues resulting in serious safety concerns. Therefore, TCR therapy may be a more promising alternative for targeting solid tumors, recognizing a wider repertoire of intracellular tumor antigens such as neoantigens. Interestingly, Harari and colleagues have recently described a new method that enables sensitive identification of rare tumor neoantigens and isolation of their cognate TCR expressed by TILs for personalized engineered T cell therapy of solid tumors [216]. Altogether, these strategies highlight the critical role of CD4+ T cells during antitumor response and support the importance of these subsets for effective T cell therapy.

## 4. Prospects of Combining Therapies for CD4^+^ T Cell Harnessing

For primary tumors, the most currently used therapy includes chemotherapy (CT) and radiotherapy (RT) able to strongly modify local immune infiltrate [217,218]. In addition to the considerable focused efforts at understanding how CT potentiates CD8^+^ T cell responses, drives Treg-mediated immune suppression and enhances antigen presentation, there is mounting evidence demonstrating the impact of CT on CD4^+^ T cell response modulation. Early reports have demonstrated, in various preclinical models, how cyclophosphamide (CTX) results in the development of robust antitumor responses associated with Th1 antitumor immunity, which induces Th2 to Th1 cytokine profile shift and polyfunctional Th1 effector cell differentiation and activation [13,219,220]. Moreover, we have previously reported that, among NSCLC patients with controlled disease after CT, patient groups presenting positive TERT-Th1 responses at baseline have significantly increased OS compared to the non-responders group [221], suggesting that CT increases the effector function of pre-existing CD4^+^ T immunity by promoting immunogenic cell death and, hence, improving patient survival [222]. In a second study, Galaine et al. reported an alternative immunogenic potential of oxaliplatin in metastatic colorectal cancer patients [223]. Indeed, oxaliplatin treatment results in increased expressions of TERT, COA-1 and mesothelin tumor antigens, which may give rise to immunogenic peptides recognized by CD4^+^ T cells.

In addition to chemotherapy, focal RT has been commonly used on its own or in association with other treatments for local tumor control. Despite its former reputation as being immunosuppressive by increasing resistant-Treg cells tumor infiltration [13,219,220], it became evident that RT can reduce tumor burden by enhancing antitumor response [224]. Altogether, these findings highlight the positive effect of CT and RT on CD4^+^ T cell modulation resulting in antitumor immunity stimulation. More recently, we have reported that CT combined with RT establishes a highly inflamed and Th1-polarized immune signature in the TME of patients and mice models [225].

Given exciting advances in immunotherapy, a combinatorial approach involving CT and RT may be a promising choice for strengthening antitumor response and for understanding their immunomodulatory effect on CD4^+^ T subsets. Indeed, rudimental reports in solid tumor patients have offered promising clinical evidence of the RT immunostimulatory capacity linked to abscopal responses [226,227]. In melanoma cancer patients treated with RT combined with anti-PD1 single dose immunotherapy, they presented Th1 immune response activation and complete clinical response [228]

More recent strategies based on RT combination with other therapies supported radiation’s role in inducing antitumor immunity in patients with metastatic melanoma and renal cell carcinoma [229,230,231,232]. A recent study in rectal cancer model has shown that RT in combination with anti-CD25/CTLA-4 was able to decrease Treg cells, improve overall survival rate and generate abscopal effect [233]. In addition, Lhuillier et al. have demonstrated that RT boosts the expression of genes encoding immunogenic neoepitopes and elicits CD8^+^ and CD4^+^ T cells responses in triple-negative breast cancer mice model. Moreover, neoantigen specific CD4^+^ T cells were able to produce Th1 cytokines, kill irradiated tumor cells and promote epitope spreading, which highlight the critical implication of RT as a combinatorial partner to neoantigen vaccination to improve vaccine efficacy [234].

The current challenge consists of modulating CD4^+^ T cell polarization and promoting appropriate antitumor CD4^+^ helper T cell recruitment within the tumor. Given the important role of epigenetics on CD4^+^ T cell differentiation and plasticity, help can be exploited at this level to overcome immunotherapy failure [235]. In murine models, Goswami et al. have reported that genetic depletion of EZH2 and its pharmacological inhibition using CPI-1505 resulted in phenotypic and functional alterations of Tregs as well as increased infiltration of Th1 and cytotoxic effector T cells. In addition, EZH2 inhibition potentiates cancer response to anti-CTLA-4 [236] and anti-PD1 [237] therapies. In hepatocellular carcinoma mice model, HDACi belinostat and anti-CTLA-4 combination increases IFN-γ tumor-reactive CD8^+^ T cell production and decreases splenic Treg cells number [238], however in syngeneic tumor mice models, HDACi CG-745 and anti-PD1 treatment increases effector helper and cytotoxic T cells proliferation while inhibiting that of Treg cells [239].

In addition to being epigenetic, T cell subset activation and differentiation are highly dependent on metabolic status [240,241], which is crucial for their antitumor functions. Recently, the CTLA-4 blockade has been shown to promote metabolic fitness and the infiltration of immune cells, especially in glycolysis-low tumors where tumor-specific CD8^+^ T cell responses correlated with phenotypic and functional destabilization of Tregs towards IFN-γ and TNF-α-producing cells in the tumor in mice model [242].

Overall, combination immunotherapy approaches involving CT, RT, epigenetic and/or metabolism mechanisms could represent the most exciting combinatorial approach for optimal targeting of CD4+ T cells to address the complexity of cancer immunopathogenesis and overcome key impediments that hamper antitumor responses.

## 5. Conclusions

Improved understanding of the critical implication of CD4^+^ T cells in antitumor immune response has undermined the classical notion of enhancing only CTL with antitumor activity to induce tumor rejection and improve cancer patients’ survival. In this review, we highlighted the critical implication of CD4^+^ T cells in antitumor immune responses. We reported recent evidence of the influence of immunotherapy to induce stronger antitumor efficacy when CD4^+^ T cell responses were reinvigorated. Moreover, conventional therapies have shown strong immunomodulatory effects on CD4^+^ T cell responses by promoting Th1 antitumor immunity and depleting immunosuppressive Treg subsets.

## 6. Future Directions

There is a growing interest in understanding the role of conventional therapies on CD4^+^ T cell modulation and the combination of immunotherapy approaches to improve antitumor immunity. Additionally, insights into the modulation of CD4^+^ T cell polarization targeting epigenetic mechanisms could be exploited to promote antitumor CD4^+^ T subsets. Likewise, a promising strategy based on metabolic modulation in order to boost effector T cells and suppress Treg differentiation seems beneficial for a combination with other immunotherapies to overcome its limitations [243,244].

Overall, combination immunotherapy approaches involving CT, RT, epigenetic and/or metabolism mechanisms could represent the most exciting combinatorial approach for optimal targeting of CD4+ T cells to address the complexity of cancer immunopathogenesis and overcome key impediments that hamper antitumor response. There is, however, still a long way to go on how to best combine available therapies, especially in the field of T cell ACT in solid cancers.

## Figures and Tables

**Table 1:** CD4^+^ T cell subsets: transcription factor, secretomes, functions and impact on the tumor growth.

Subsets	Transcription Factors	Secretome	Functions		Impact on Tumor Growth
			Antitumoral	Protumoral	
**Th1**	T-bet^+^	IFNγ,IL-2, TNF	-Stimulate CTL and NK cell functions [16,17,18] -Enhance MHC-I and II expression by tumor cells [19,20] -Promote tumor effector T cell infiltration (CXCL9 and CXCL10 secretion by TAM and CAF) [21,22]		Inhibition
**Th2**	GATA3^+^ IRFA4^+^	IL-4, IL-5, IL-9,IL-13	-Induce M2 macrophage infiltration and cancer eradication [23] -NK stimulation [24,25,26]	-Promote B cell activation and IL-10 production [27,28]	Promotion
**Th9**	IRF4^+^ PU.1^+^	IL-9	-Promote antigen presentation by DC leading to D8+ T cell activation [29] -Present cytolytic activity [30]	-Promote tumor metastasis [31]	Ambivalent
**Th17**	IRF4^+^ RORyt^+^	IL-17 IL-21 IL-22	-Induce apoptosis of cancer cells (IL17 receptor ligation) [32] -Enhance antitumor macrophage polarization and cytotoxic markers expression on NK [33,34] -Recruit and activate T cells, DC and NK cells [33,35]	-Stimulate tumor cell proliferation and cancer invasion and metastasis [36,37] -Promote angiogenesis [38,39] -Recruit MDSC [40]	Ambivalent
**Treg**	FOXP3	IL10 TGFβ		-Inhibit antitumor immunity through immune suppressive mechanisms [41,42]	Promotion
**CD4^+^CTL**		GZM PFN	-Induce tumor cells eradication [12,43,44,45,46,47,48,49]		Inhibition
**GC Tfh** **cTfh**	BCL6^+^ BCL6^-^	IL-21 IL-4 IL-21 IL-10 IL-2	-Interaction with B cells enhance GZMB expression [50]		Inhibition

Abbreviations: Th: T helper; Treg: regulatory CD4^+^ T cell; CTL: cytotoxic T cell; GC-Tfh: germinal center T follicular helper cell; c-Tfh: circulating follicular helper cell; NK: natural killer; DC: dendritic cell; TAM: tumor associated macrophage; CAF: cancer associated fibroblast; MDSC: myeloid-derived suppressor cell; T-bet: T-box expressed in T cells; GATA3: GATA binding protein 3; IRF4: interferon regulatory factor 4; PU.1: purine-rich box 1; RORγt: RAR related orphan receptor gamma; FOXP3: forkhead protein box O4; BCL6: B-cell lymphoma 6; Secretome abbreviations: IL: interleukin; IFNγ: interferon gamma; TNF: tumor necrosis factor; TGFβ: transforming growth factor β; GZM: granzyme; PFN: perforin.
